# Long-Term Oncologic Outcomes for Patients Undergoing Volatile Versus Intravenous Anesthesia for Non-Small Cell Lung Cancer Surgery: A Retrospective Propensity Matching Analysis

**DOI:** 10.1177/1073274818775360

**Published:** 2018-05-09

**Authors:** Tak Kyu Oh, Kwhanmien Kim, Sanghoon Jheon, Jaebong Lee, Sang-Hwan Do, Jung-Won Hwang, In-Ae Song

**Affiliations:** 1 Department of Anesthesiology and Pain Medicine, Seoul National University Bundang Hospital, Bundang-gu, Seongnam, Korea; 2 Department of Thoracic Surgery, Seoul National University Bundang Hospital, Bundang-gu, Seongnam, Korea; 3 Medical Research Collaborating Center, Seoul National University Bundang Hospital, Bundang-gu, Seongnam-si, Gyeonggi-do, Korea

**Keywords:** cancer, cancer prevention, cancer survival, cancer treatment, NSCLC

## Abstract

Propofol-based total intravenous anesthesia (TIVA) has been reported to improve long-term outcome following cancer surgery, when compared with inhalation agents. However, such investigational reports are still controversial, and no studies have been conducted in relation to non-small cell lung cancer (NSCLC) surgery. The present study aimed to compare the favorable effects of TIVA versus inhalation agents on recurrence-free survival and overall survival after curative resection of NSCLC. This retrospective cohort study examined medical records of the patients who were diagnosed with NSCLC and underwent curative resection at Seoul National University Bundang Hospital from August 2003 to July 2012. The primary outcome included the comparison of postoperative overall survival and recurrence-free survival in both groups. To balance the 2 groups for analysis, a propensity matching method was used, and stratified Cox proportional hazard models were used for statistical analysis. This study included 943 cases of NSCLC for final analysis, and the cases were divided into the TIVA group (n = 749) and inhalation group (n = 194). Propensity matching produced 196 patients in each group. The final analysis revealed no significant difference in the hazard ratio (HR) for recurrence between the TIVA and inhalation groups (*P* = .233). The HR for death between the 2 groups was not significantly different either (*P* = .551). In this study, we found no benefit of propofol-based TIVA for long-term oncologic outcome after NSCLC surgery, relative to inhalation agents.

## Introduction

Lung cancer is one of the leading causes of cancer-related death worldwide.^1^ In particular, non-small cell lung cancer (NSCLC) is the most common type of lung cancer, accounting for 80% of total lung cancer diagnoses. Curative resection is known as a treatment option for long-term survival.^2^ However, the long-term survival rate after curative resection for NSCLC has been reported to be less than 50%, with 33.1% of patients with NSCLC reportedly having recurrence within 2 years.^3^

To perform curative resection for NSCLC, general anesthesia is usually provided by using an anesthetic comprising either propofol or inhalation agents. The effects of these 2 anesthetic techniques on long-term oncologic outcome have been addressed in preclinical studies.^4,5^ Inhalation agents have been reported to suppress natural killer cell activity and promote tumor metastasis. More recently, a retrospective study involving more than 7000 patients reported that the long-term survival after curative resection in a propofol-based total intravenous anesthesia (TIVA) group outpaced that of the inhalation agent group.^6^ Another retrospective study conducted in patients who received propofol-based TIVA after surgery for esophageal cancer also showed better overall survival (OS) and recurrence-free survival (RFS), compared with an inhalation-based anesthetic group.^7^

However, no attempt has been made to assess the effects of inhalation agents and TIVA on the outcomes of NSCLC treated with surgery alone. This study aimed to compare RFS and OS between the choice of propofol-based TIVA and inhalation agents for general anesthesia when treating NSCLC with curative resection.

## Materials and Methods

This retrospective study was approved by the institutional review board of the Seoul National University Bundang Hospital (SNUBH; approval number: B-1708/412-133). Because this was a retrospective review of electronic patient medical records, the requirement for informed consent was waived.

### Inclusion of Patients

Medical records of patients aged 19 years or older, who were diagnosed with NSCLC and underwent elective curative resection (lobectomy, segmentectomy, and wedge resection), between August 2003 and July 2012, were examined. The exclusion criteria were (1) intraoperative conversion to pneumonectomy or bilobectomy, (2) pathologic staging M1 or N3, (3) incomplete resection, (4) loss to follow-up within 5 years postsurgery, (5) death within 1 month due to surgery-related complications, (6) occurrence of other primary cancers within 5 years after surgery, and (7) incomplete medical records. Lobectomy with sublobar resection in other lobes was considered as lobectomy, and segmentectomy with wedge resection in other lobes was considered as segmentectomy.

### Anesthetic Technique for Lung Cancer Surgery in SNUBH

Patients were divided into groups receiving either inhalation-based agents (inhalation group) or propofol-based TIVA (TIVA group), depending on the anesthetics chosen by anesthetists at their discretion during the study period. For the thoracic anesthesia used during the lung cancer surgery, sevoflurane was administered to those in the inhalation group and continuous propofol infusion was administered using a target control infusion system to those in the TIVA group. In both groups, intravenous (IV) remifentanil continuous infusion was also performed. Although the anesthetics used were different (inhalation agent vs propofol), general care for the patients was consistent in both groups. Epidural anesthesia or analgesia was not performed during the study period.

### Measurements

The following patient information was collected: age, sex, body mass index (kg/m^2^), American Society of Anesthesiologists classification, histologic tumor type, surgery type (video-assisted thoracic surgery), preoperative comorbidities (hypertension, diabetes mellitus, stroke, ischemic heart disease), pathologic tumor stage, pathologic lymph node stage, adjuvant chemotherapy or adjuvant radiotherapy, surgery time (minutes) and anesthesia time (minutes), total opioid dosage in postoperative days 0 to 3, the date of death and date of recurrence, and intraoperative anesthetics used. Tumor stage and lymph node status were based on the American Joint Committee on Cancer Seventh Edition guidelines.^8^ Preoperative hypertension and diabetes mellitus were determined by regular intake of related medication before surgery, and a history of ischemic heart disease included stable or unstable angina and myocardial infarction. Total opioid dosage on postoperative days 0 to 3 was calculated and combined according to a standard conversion ratio.^9^ The date of death was set under the approval of the Ministry of the Interior and Safety in South Korea, and the date of recurrence was the date on which the respective patients were diagnosed with a recurrence during an outpatient clinic follow-up visit.

### Clinical Outcome

The comparison of RFS and OS after lung cancer surgery between the TIVA and inhalation groups was used as the primary outcome measure. The OS was defined as the period from surgery date to the date of death, and RFS was defined as the period from surgery date to the date of recurrence or death.

### Statistical Methods

In the comparison of the TIVA and inhalation groups, the *t* test was used for continuous variables and the χ^2^ test was used for categorical variables. To achieve balance for all intergroup covariates with a standardized mean difference (SMD) of less than 0.1, propensity score (PS) matching was performed.^10^ Univariate regression analysis was performed to identify covariates that individually influence recurrence or death after lung cancer surgery. Finally, after achieving covariate balance between the 2 groups with PS matching, we analyzed the data using a stratified Cox proportional hazard model. The results of the stratified Cox regression analysis were presented as hazard ratios (HRs) and 95% confidence intervals. In addition, the TIVA and inhalation groups were compared for OS and RFS using the Kaplan-Meier method and tested using the log-rank test. R software (version 3.3.2; R Development Core Team, Vienna, Austria) was used for all statistical analyses. A *P* value <.05 was considered statistically significant.

## Results

### Inclusion of Patients

**Figure 1.**
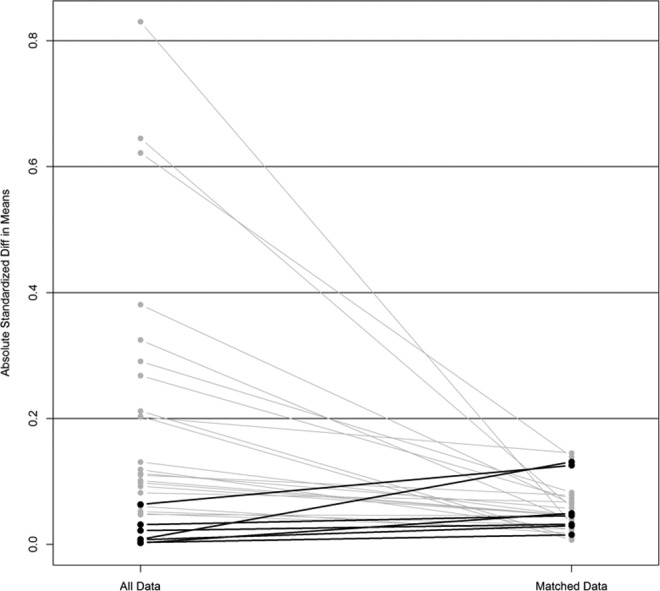
Balance of covariates before and after propensity score matching.

A total of 1548 patients were diagnosed with NSCLC and underwent elective lung cancer surgery between August 2003 and July 31, 2012. Patients were excluded from the analysis owing to the following reasons: (1) intraoperative conversion to bilobectomy or pneumonectomy (n = 82), (2) incomplete resection (n = 86), (3) loss to follow-up within 5 years (n = 82), (4) death within 1 month due to postoperative complications (n = 3), (5) pathologic stage of N3 or M1 (n = 49), (6) occurrence of other primary cancers rather than recurrence within 5 years after surgery (n = 89), and (7) incomplete medical records (n = 131). This study included 943 patients for analysis, with 749 patients in the TIVA group and 194 patients in the inhalation group. The differences in the baseline characteristics between the TIVA and inhalation groups are shown in Table 1. A total of 181 patients in each group were selected from PS matching analysis. The SMDs for all the covariates were less than 0.1, indicating a good balance (*P* > .1; Figure 1).

**Table 1.** Baseline Characteristic Before and After Propensity Score Matching.^a^

	Before Matching				After Matching			
	TIVA (n = 749)	Inhalation (n = 194)	*P* Value	SMD	TIVA (n = 181)	Inhalation (n = 181)	*P* Value	SMD
Female	285 (38.1)	65 (32.5)	.177	0.117	64 (35.4)	62 (34.3)	.912	0.023
Age (years)	63.3 (10.0)	63.9 (10.6)	.527	0.050	63.3 (10.9)	63.5 (10.4)	.820	0.024
BMI (kg/m^2^)	23.9 (2.8)	23.5 (2.8)	.110	0.127	23.9 (2.8)	23.6 (2.8)	.849	0.019
ASA (%)			<.001	0.457			.691	0.091
1	213 (28.4)	44 (22.7)			50 (27.6)	44 (24.3)		
2	477 (63.7)	103 (53.1)			98 (54.1)	99 (54.7)		
3	59 (7.9)	47 (24.2)			33 (18.2)	38 (21.0)		
Histology (%)			.498	0.094			.789	0.072
Squamous cell carcinoma	175 (23.4)	53 (27.3)			45 (24.9)	48 (26.5)		
Adenocarcinoma	478 (63.8)	116 (59.8)			117 (64.6)	111 (61.3)		
Other^b^	96 (12.8)	25 (12.9)			33 (18.2)	38 (21.0)		
Non-VATS (%)	255 (34.0)	72 (37.1)	.474	0.094	57 (31.5)	68 (37.6)	.269	0.128
Type of surgery (%)			.718	0.064			.947	0.035
Lobectomy	656 (87.6)	166 (85.6)			159 (87.8)	159 (87.8)		
Segmentectomy	26 (3.5)	7 (3.6)			6 (3.3)	7 (3.9)		
Wedge resection	67 (8.9)	21 (10.8)			16 (8.8)	15 (8.3)		
Preoperative hypertension (%)	147 (19.6)	29 (14.9)	.165	0.124	27 (14.9)	24 (13.3)	.763	0.048
Preoperative DM (%)	64 (8.5)	12 (6.2)	.353	0.090	11 (6.1)	9 (5.0)	.818	0.048
Preoperative stroke history (%)	36 (4.8)	9 (4.6)	1.000	0.008	11 (6.1)	6 (3.3)	.320	0.131
Preoperative IHD history (%)	31 (4.1)	20 (10.3)	.001	0.240	21 (14.9)	13 (7.2)	.207	0.152
Preoperative COPD history (%)	26 (3.5)	6 (3.1)	.970	0.021	4 (2.2)	5 (2.8)	.100	0.035
Tumor (%)			.471	0.120			.920	0.074
0-1	370 (49.4)	97 (50.0)			88 (48.6)	88 (48.6)		
2	299 (39.9)	73 (37.6)			74 (40.9)	70 (38.7)		
3	52 (6.9)	12 (6.2)			10 (5.5)	12 (6.6)		
4	28 (3.7)	12 (6.2)			9 (5.0)	11 (6.1)		
Node (%)			.449	0.106			.852	0.060
0	540 (72.1)	146 (75.3)			132 (72.9)	136 (75.1)		
1	115 (15.4)	30 (15.5)			29 (16.0)	28 (15.5)		
2	94 (12.6)	18 (9.3)			20 (11.0)	17 (9.4)		
Adjuvant radiotherapy (%)	615 (82.1)	163 (84.0)	.604	0.051	152 (84.0)	154 (85.1)	.884	0.031
Adjuvant chemotherapy (%)	686 (91.6)	182 (93.8)	.383	0.086	172 (95.0)	170 (93.9)	.818	0.048
Year at surgery			<.001	0.654			.379	0.147
2003-2006	138 (18.4)	25 (12.9)			20 (11.0)	25 (13.8)		
2007-2009	279 (37.2)	131 (67.5)			117 (64.6)	122 (67.4)		
2010-2012	332 (44.3)	38 (19.6)			44 (24.3)	34 (18.8)		
Intraoperative pRBC transfusion	77 (10.3)	47 (24.2)	<.001	0.376	37 (20.4)	40 (22.1)	.797	0.041
Morphine equivalent consumption in POD 0 to 3	101.2 (75.1)	116.6 (76.1)	.011	0.204	117.9 (77.2)	117.4 (77.0)	.948	0.007

Abbreviations: ASA, American Society of Anesthesiologists; BMI, body mass index; COPD, chronic obstructive pulmonary disease; DM, diabetes mellitus; IHD, ischemic heart disease; POD, postoperative day; pRBC, packed red blood cell; SD, standard deviation; SMD, standardized mean difference; TIVA, total intravenous anesthesia; VATS, video-assisted thoracic surgery.

^a^ Presented as mean (standard deviation) or number (percentage).

^b^ Others: large cell type, sarcomatoid lung cancer.

### Stratified Cox Regression Analysis After PS Matching Between the TIVA Group and Inhalation Group

**Figure 2.**
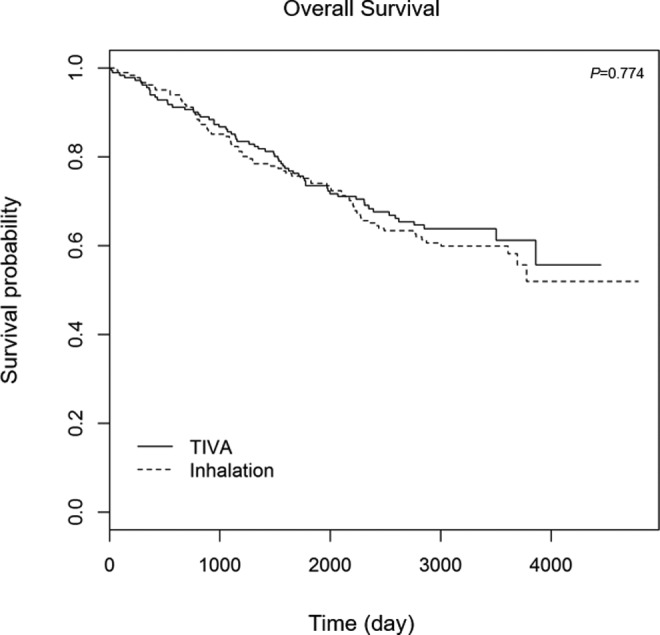
Overall survival after lung cancer surgery between the inhalation and TIVA groups after propensity score matching. TIVA indicates total intravenous anesthesia.

**Figure 3.**
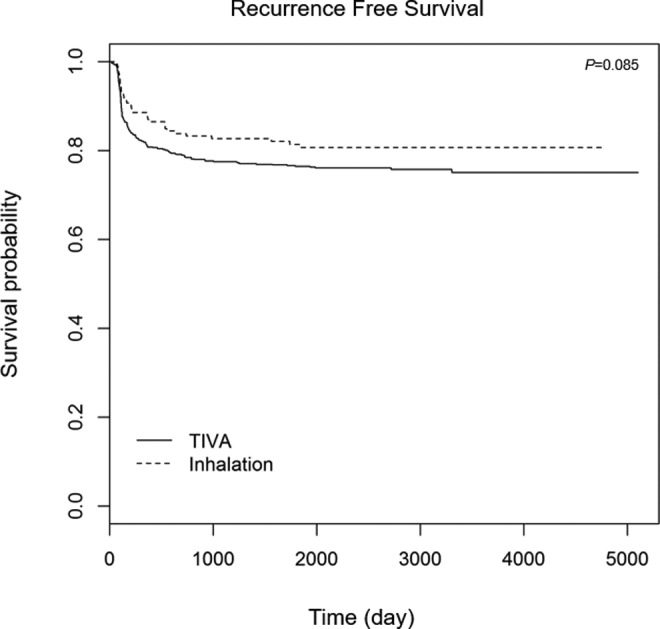
Recurrence-free survival after lung cancer surgery between the inhalation and TIVA groups after propensity score matching. TIVA indicates total intravenous anesthesia.

The results of the univariate Cox regression analysis to identify variables for death or recurrence after surgery are shown in Table 2. The results of the Cox proportional hazard model for recurrence and death before and after PS matching are listed in Table 3. No significant difference was found in the HR for recurrence between the TIVA and inhalation groups before and after PS matching (*P* = .111 before PS matching and *P* = .233 after PS matching). The HR for death showed no significant difference between the 2 groups before and after PS matching (*P* = .260 before PS matching and *P* = .551 after PS matching). The Kaplan-Meier curves of RFS and OS after PS matching are illustrated in Figures 2 and 3. In the Kaplan-Meier curve after PS matching, the differences in OS (*P* = .774) and RFS (*P* = .085) were not significant between the 2 groups.

**Table 2.** Univariate Logistic Analysis for Recurrence and Death After Lung Cancer Surgery.

Variable	Recurrence	95% Confidence Interval			Death	95% Confidence interval		
	HR	Lower	Upper	*P* Value	HR	Lower	Upper	*P* Value
Sex								
Male	1.000				1.000			
Female	0.839	0.632	1.115	.227	0.515	0.405	0.657	<.001
Age, years	1.004	0.991	1.017	.571	1.046	1.033	1.058	<.001
Body mass index, kg/m^2^	1.038	0.989	1.089	.132	0.934	0.899	0.971	.001
ASA								
1	1.000				1.000			
2	1.437	1.030	2.003	.033	1.416	1.084	1.849	.011
3	1.248	0.757	2.059	.385	2.142	1.513	3.033	<.001
Histology								
Squamous cell	1.000				1.000			
Adenocarcinoma	0.704	0.525	0.945	.019	0.565	0.447	0.713	<.001
Others^a^	0.299	0.162	0.553	<.001	0.690	0.490	0.971	.033
Type of operation I								
VATS	1.000				1.000			
Open thoracotomy	2.233	1.705	2.925	<.001	2.037	1.644	2.524	<.001
Type of operation II								
Lobectomy	1.000				1.000			
Segmentectomy	0.000	0.000		.992	0.270	0.101	0.725	.009
Wedge resection	0.332	0.164	0.673	.002	0.593	0.382	0.923	.021
Preoperative hypertension	1.102	0.786	1.545	.572	0.898	0.675	1.195	.460
Preoperative diabetes mellitus	1.209	0.763	1.917	.419	1.055	0.712	1.564	.788
Preoperative stroke history	0.655	0.308	1.392	.271	0.843	0.484	1.468	.546
Preoperative IHD history	0.778	0.399	1.516	.461	1.335	0.858	2.079	.200
Preoperative COPD history	0.623	0.256	1.512	.295	1.125	0.679	1.866	.648
Tumor								
0-1	1.000				1.000			
2	3.986	2.808	5.658	<.001	2.170	1.706	2.762	<.001
3	7.711	4.849	12.264	<.001	3.723	2.565	5.404	<.001
4	8.803	5.256	14.744	<.001	5.578	3.778	8.237	<.001
Node								
0	1.000				1.000			
1	75.275	41.354	137.018	<.001	2.133	1.637	2.778	<.001
2	100.4422	54.814	184.053	<.001	2.924	2.222	3.849	<.001
Adjuvant radiotherapy	0.308	0.232	0.408	<.001	0.309	0.246	0.387	<.001
Adjuvant chemotherapy	0.632	0.416	0.960	.031	0.816	0.577	1.152	.248
Surgery time (minutes)	1.004	1.002	1.005	<.001	1.004	1.003	1.005	<.001
Anesthesia time (minutes)	1.004	1.003	1.006	<.001	1.004	1.003	1.005	<.001
Years at surgery								
2003-2006	1.000				1.000			
2007-2009	0.952	0.659	1.374	.792	0.912	0.695	1.196	.505
2010-2012	0.882	0.603	1.289	.516	0.660	0.484	0.899	.009
Intraoperative pRBC transfusion	1.460	1.020	2.089	.039	1.606	1.224	2.107	.001
Postoperative complication	1.014	0.600	1.714	.959	1.393	0.966	2.009	.076
Clavien-Dindo classification								
None	1.000				1.000			
I, II	1.124	0.627	2.013	.695	1.392	0.916	2.115	.121
IIIA, IIIB	0.369	0.092	1.485	.160	1.033	0.512	2.084	.928
IVA, IVB	0.815	0.114	5.812	.838	0.969	0.241	3.897	.965

Abbreviations: ASA, American Society of Anesthesiologists; COPD, chronic obstructive pulmonary disease; HR, hazard ratio; IHD, ischemic heart disease; RBC, red blood cell; VATS, video-assisted thoracic surgery.

^a^ Others: Large cell type, sarcomatoid lung cancer.

**Table 3.** Cox Proportional Hazard Model for Recurrence and Death After Lung Cancer Surgery.

Model	Recurrence	95% CI		*P* Value	Death	95% CI		
	HR	Lower	Upper		HR	Lower	Upper	*P* Value
Unadjusted								
Inhalation	1.000				1.000			
TIVA	1.339	0.935	1.916	0.111	0.867	0.677	1.111	0.260
Matched (stratified Cox regression)								
Inhalation	1.000				1.000			
TIVA	1.310	0.841	2.041	0.233	0.902	0.643	1.265	0.551

Abbreviations: CI, confidence interval; HR, hazard ratio; TIVA, total intravenous anesthesia.

## Discussion

The present study demonstrated that the anesthetic technique for administering the TIVA and inhalation agents did not have effects on OS or RFS, which were long-term outcome measures of curative resection for NSCLC. These results are in contrast with those from recent previous retrospective studies.^6,7,11,12^ The results of this study revealed that TIVA is not an advantageous anesthetic agent for lung cancer surgery over inhalation-based agents. In addition, this study confirms the controversy involving which anesthetic technique is effective in improving oncologic outcome of cancer.

The contrasting results found in this study can be attributed to the fact that we focused on a single cancer type (NSCLC). When compared with cohort studies using patients with different types of cancer,^6,12^ the TIVA used for patients with NSCLC may have different effects on long-term oncologic outcomes. Given the fact that curative resection of NSCLC is characterized by poor OS and RFS,^3^ it is difficult to assess the antitumor effects of propofol-based TIVA using a small population.^13^ Therefore, we suggest that the significant clinical effects of TIVA may not have been identified given the design and sample size of this study. Second, in this study, the inhalation group was administered 1.5 to 2.5 mg of 1% propofol/kg of body weight to induce a pleasant loss of consciousness in the early stage of general anesthesia. Although no further propofol was administered to the inhalation group, the initial injection of propofol may have influenced the results of this study. If volatile induction and maintenance of anesthesia (VIMA) had been performed for the inhalation group, the results may have been different.

A recent study investigating surgery for esophageal cancer reported that TIVA was more effective in improving RFS and OS than inhalation-based agents,^7^ which is noteworthy since the study had a similar design to our study, but had contrasting results. The significant difference between the 2 studies lies in the fact that epidural analgesia was not utilized in the present study, but was in the previous study. The use of perioperative epidural anesthesia or analgesia alone has been established as an important factor affecting long-term oncologic outcome of cancer.^14^ If esophageal cancer or lung cancer surgery requires a thoracotomy, general anesthesia can be combined with an epidural analgesia. As a result, the stress response is reduced and immune dysfunction is also minimized.^15^ Therefore, it is possible that the previous study^7^ used a small dose of IV opioid during surgery, although it was not specifically described. However, no epidural analgesia was used in this study due to possible complications associated with epidural catheterization. Since a high dose of opioid can influence the long-term outcome after NSCLC surgery,^16^ this is an important issue to bear in mind.

In addition to not using epidural analgesia in our study, the continuous infusion of remifentanil in both groups could be another important reason for our negative outcome. Assuming the immunosuppression was caused by the opioid,^17^ intraoperative infusion of remifentanil may have affected outcomes regarding OS or RFS after lung cancer surgery. Although we matched the total opioid use for 3 days after surgery to balance the 2 groups using a standard conversion ratio, we did not consider the dosage of remifentanil during surgery. However, we reported recently that remifentanil dosage during surgery was not clinically associated with OS or RFS in esophageal cancer surgery.^18^ In summary, issues regarding the impact of epidural analgesia or opioid use on OS or RFS in lung cancer are still questionable. Therefore, the results of a prospective ongoing clinical trial (NCT01179308) will be important in the future.

Lastly, the previous study^7^ did not state which IV drug was used as the induction IV agent in the early stage. As mentioned earlier, in the present study, 1.5 to 2.5 mg of 1% propofol/kg of body weight was used for the inhalation group to induce general anesthesia. Given that anesthetic agents, such as 1% propofol or thiopental sodium, can be used instead of VIMA to induce anesthesia in adult patients receiving inhalational anesthesia, the drug used as the induction IV agent in the previous study^7^ could cause the difference in results between the 2 studies. Finally, the surgical time of esophageal surgery is longer than that of lung cancer surgery; therefore, the exposure to propofol was also longer in esophageal cancer surgery. However, this is still a controversial issue and requires more clinical studies.

This study has several limitations. First, as commonly found in single-center retrospective observational studies, selection bias may exist along with a possible lack of generalizability of the results. Second, since the study was based on data from a 9-year period, there could have been changes in surgical or patient management. However, these factors were not adequately considered. Third, a small sample size of patients was included in our study, which may account for our negative finding. Finally, as mentioned before, VIMA using anesthetic gas only was not provided to the inhalation group. Nonetheless, this study was the first to analyze the effects of TIVA compared to inhalation-based agents in terms of OS and RFS after NSCLC surgery and thus is meaningful.

In conclusion, our study showed no better benefit for propofol-based TIVA, in comparison with inhalation agents, in terms of long-term oncologic outcome after NSCLC surgery. Thus, this study confirms the existing controversy over an optimal anesthetic management for lung cancer surgery and suggests the need for further well-designed prospective studies.
